# An Endolysin LysSE24 by Bacteriophage LPSE1 Confers Specific Bactericidal Activity against Multidrug-Resistant *Salmonella* Strains

**DOI:** 10.3390/microorganisms8050737

**Published:** 2020-05-15

**Authors:** Yifeng Ding, Yu Zhang, Chenxi Huang, Jia Wang, Xiaohong Wang

**Affiliations:** 1 Key Laboratory of Environment Correlative Dietology, Huazhong Agricultural University, Wuhan 430070, China; yifengding@webmail.hzau.edu.cn (Y.D.); wangjia@mail.hzau.edu.cn (J.W.); 2 College of Food Science and Technology, Huazhong Agricultural University, Wuhan 430070, China; 123zy@webmail.hzau.edu.cn (Y.Z.); ch679@cornell.edu (C.H.)

**Keywords:** endolysins, bacteriophages, *Salmonella*, purification, antimicrobial activity

## Abstract

*Salmonella* is responsible for a wide range of infections and is a constant threat to public health, particularly in light of emerging antibiotic resistance. The use of bacteriophages and phage endolysins as specific antibacterial agents is a promising strategy to control this bacterial infection. Endolysins are important proteins during the process of bacteria lysis by bacteriophages. In this study, we identify a novel endolysin, named LysSE24. LysSE24 was predicted to possess *N*-acetylmuramidases activity, with a molecular mass of ca. 17.4 kDa and pI 9.44. His-tagged LysSE24 was heterologously expressed and purified by Ni-NTA chromatography. LysSE24 exhibited optimal bactericidal activity against *Salmonella* Enteritidis ATCC 13076 at a concentration of 0.1 μM. *Salmonella* population (measured by OD_600 nm_) decreased significantly (*p* < 0.05) after 10 min of incubation in combination with the outer membrane permeabilizer in vitro. It also showed antibacterial activity against a panel of 23 tested multidrug-resistant *Salmonella* strains. Bactericidal activity of LysSE24 was evaluated in terms of pH, temperature, and ionic strength. It was very stable with different pH (4.0 to 10.0) at different temperatures (20 to 60 °C). Both K^+^ and Na^+^ at concentrations between 0.1 to 100 mM showed no effects on its bactericidal activity, while a high concentration of Ca^2+^ and Mg^2+^ showed efficacy. Transmission electron microscopy revealed that exposure to 0.1 μM LysSE24 for up to 5 min caused a remarkable modification of the cell shape of *Salmonella* Enteritidis ATCC 13076. These results indicate that recombinant LysSE24 represents a promising antimicrobial activity against *Salmonella*, especially several multidrug-resistant *Salmonella* strains. Further studies can be developed to improve its bactericidal activity without the need for pretreatment with outer membrane-destabilizing agents by synthetic biology methods.

## 1. Introduction

*Salmonella* bacteria is the etiologic agent of salmonellosis for humans in many countries for at least over 100 years [1]. Salmonellosis symptoms include nausea, vomiting, abdominal pain, diarrhea, fever, and headache. *Salmonella* infection can be detected in various foods, including, but not limited to, eggs and egg products, meats, dairy products, fresh fruits, and vegetables [2]. It accounts for 93.8 million cases of foodborne illness and 155,000 deaths in industrialized and underdeveloped countries per year [3]. In the US, it is estimated that salmonellosis cause 1.2 million people with 23,000 hospitalizations and 450 deaths annually [4]. In China, approximately 70 cases of salmonellosis were reported from 2008 to 2012, which led to 4151 hospitalized cases and four deaths in many provinces [5,6,7]. To date, more than 2600 *Salmonella* serovars have been reported. Of these, the most common serotypes associated with human illness are *Salmonella enterica* serovar Typhimurium (*S*. Typhimurium) and *S*. *enterica* serovar Enteritidis (*S*. Enteritidis) [2,8,9,10]. The routine use of antimicrobial agents has been considered as the most effective intervention strategy to eliminate *Salmonella* contamination. However, the recent rise of multidrug-resistant *Salmonella* strains leads to a more serious threat to public health, and thus makes developing novel antibiotic alternatives imperative.

Bacteriophages are viruses that infect bacteria. They have been optimized by millions of years of evolution to specifically recognize and effectively kill their target cells [11]. In recent years, the use of lytic bacteriophages has become an option in treating emerging *Salmonella* serotypes or antimicrobial-resistant *Salmonella*. The use of phages as biocontrol agents provides several advantages over traditional antibiotic treatment, such as its target specificity without damage to coexisting microflora, inherent low toxicity, robustness to harsh environments, widespread distribution, self-replication, and cheap preparation [12,13]. In addition, bacteriophage endolysins with host specificity and host lysis activities might be preferred to food applications rather than phages [14].

Endolysins (lysins) are bacteriophage-encoded enzymes that degrade specific bonds within the bacterial cell wall, resulting in the release of progeny virions [15,16]. Endolysins are peptidoglycan hydrolases (PGH) and typically differ between Gram-positive and Gram-negative bacteria due to differences in the bacterial cell wall structure. Generally, endolysins targeting Gram-positive bacteria often have a modular structure that includes two conserved protein domains, the *N*-terminal enzymatic activity domain (EAD) and the C-terminal cell wall binding domain (CBD). Five types of EADs have been reported according to the cleavage sites: *N*-aceytlmuramidases (endolysins), *N*-acetyl-b-d-glucosaminidases (glycosidases), *N*-acetylmuramoyl-l-alanineamidases, l-alanoyl-d-glutamate endopeptidases, and interpeptide bridge-specific endopeptidases [14,17]. Meanwhile, endolysins targeting Gram-negative bacteria exhibit a globular structure that contains a single catalytic domain [18].

Endolysins can be a novel biocontrol agent to kill target bacteria when it is applied exogenously in the form of recombinant proteins. This feature has led to a renewed interest, particularly in light of emerging and spreading resistance of bacteria against classical antibiotics [19]. In the last two decades, endolysins have proven their efficacy in controlling bacterial contamination in food [20] and combating bacterial infection in the fields of medicine [21,22]. Thus, endolysins have increased potential as effective antibacterial agents against infectious pathogens. Endolysins have multiple advantages over antibiotics. First, endolysins specifically target the peptidoglycan layer in the bacteria. So far, no emergences of resistance have been reported to date [23]. Furthermore, endolysins can be modified by genetic engineering to alter their host range and efficiency [24,25]. Finally, endolysins can be cloned into expression vectors for large scale synthesis due to their proteinaceous nature [26]. Endolysins from bacteriophages infecting Gram-positive bacteria have been well studied, but the application of endolysins derived from bacteriophages infecting Gram-negative bacteria is still at the preliminary stage. The outer membrane of Gram-negative bacteria prevents the access of endolysins to the peptidoglycan layer from outside [27,28]. However, the study of OM permeabilizers (OMPs) has aroused hope; OMPs and phage endolysins can be used to work together on Gram-negative bacteria, such as *Pseudomonas*, *E. coli,* and *Salmonella* [19]. To utilize this advantage of endolysins, further efforts and studies need to be conducted on the development of novel phage-based biocontrol agents against foodborne pathogens. Previously, *Salmonella* Enteritidis targeting bacteriophage LPSE1 was isolated from environmental water. Its genome was completely sequenced [29]. It has a circular genome consisting of 41,854 bp with a GC content of 49.83% and 32 ORFs (open reading frames). In addition, LPSE1, belonging to the *Siphoviridae* family, infects a broad range of *Salmonella* strains. In this study, an endolysin LysSE24 from the bacteriophage LPSE1 is purified, and its lysis activity is evaluated.

## 2. Materials and Methods

### 2.1. Bacterial Strains, Plasmid and Growth Conditions

The bacterial strains used in this study are described in Table 1. These include a broad range of reference strains as well as multidrug-resistant strains. All strains were stored frozen at −80 °C in 20% (*vol/vol*) glycerol and cultured in LB medium at 37 °C. Vector pET-28b, *E*. *coli* BL21, and *E*. *coli* C41 were prepared for cloning and protein expression (Novagen, USA). TSB-YE and LB medium were purchased from Hopebio (Qingdao, China). The protein markers were purchased from SMOBIO (Hsinchu City, Taiwan).

### 2.2. Bioinformatics Analysis of LysSE24

From the complete genome sequence of *Salmonella* phage LPSE1 (NCBI accession no. KY379853.1), the possible DNA sequence encoding endolysin LysSE24 (NCBI accession number: APU02985.1) was identified. The physical and chemical properties of the LysSE24 sequence, including molecular weight, isoelectric point, instability coefficient, and hydrophilic coefficient, were predicted by the online tool ProtParam https://web.expasy.org/protparam/). BLASTP was used to search the non-redundant database to find the amino acid sequence similar to LysSE24. At the same time, the sequences of *Salmonella* phage endolysins (from BLASTP) were compared by Clustal Omega (https://www.ebi.ac.uk/Tools/msa/clustalo/) and software Jalview 2.11.0. Then, the conserved domain was predicted by NCBI Conserved Domain Database and Pfam database. The secondary structure of the LysSE24 sequence was predicted by the online tool JPred online server in the software Jalview 2.11.0. The tertiary structure analysis was carried out using the Swiss-Model online server ( http://swissmodel.expasy.org). The evaluation of the tertiary structure model refers to the Ramachandran Plot by using software BIOVIA Discovery Studio 2019, and Verify-3D evaluation was carried out by using The Structure Analysis and Verification Server version 5.0 (https://servicesn.mbi.ucla.edu/Verify3D/). The tertiary structure of LysSE24 was visualized by software VMD 1.9.1.

### 2.3. Cloning, Expression, and Purification of LysSE24

The gene encoding LysSE24 was amplified by PCR using DNA of phage LPSE1 as the template. It is with a His-tag at the C-terminal. The primers were designed as forward primer: 5′-CCATATGATGTCAAACCGAAACATCAGTG-3′, reverse primer: 5′-CCTCGAGGCTACTTCGCATCGCGCCCTAC-3′, with the *Nde*I/*Xho*I restriction endonuclease sites underlined. Next, the PCR product was digested with *Nde*I and *Xho*I and then ligated into the *Nde*I- and *Xho*I-linearized vector pET-28b to construct recombinant plasmids for endolysin LysSE24 expression.

In order to optimize the expression of endolysin, it was detected in different expression strains and different temperatures. Firstly, the recombinant plasmid was transformed into *E. coli* BL21 and *E. coli* C41, respectively. A single colony of each transformant was cultured overnight in 5 mL LB medium supplemented with 50 μg/mL kanamycin (LBK) at 37 °C. After overnight, the culture was used to inoculate another 5 mL of fresh LBK, and 100 mL batch of LBK was inoculated when the OD_600 nm_ value was close to 0.6. When the culture of OD_600 nm_ was approximately 1.0, the expression of endolysin can be initiated by adding IPTG to a final concentration of 0.25 mmoL/L. Induction was conducted and attempted for 16 h with shaking at 16 °C, 4 h with shaking at 30 °C, and 4 h with shaking at 37 °C, respectively. The culture was collected by centrifugation at 7000× *g* for 20 min. The supernatant was washed three times with phosphate-buffered saline (PBS; 50 mmol/L NaH_2_PO_4_, 300 mmol/L NaCl, pH 7.5). Finally, the particles were resuspended in 40 mL PBS and ultrasonic crushed at a temperature below 10 °C (30 min 2 s on, 2 s 105 W off) using an ice bath. The supernatant (the native protein extract after sonication, NPE) and the denatured protein extract (solubilization of the pellet after sonication in 8.0 moL/L urea, DPE) were collected for the SDS-PAGE analysis. The NPE was collected and then purified with HisPur Ni-NTA spin columns according to the manufacturer’s protocol. Protein was quantitated by the Bradford method. Purified protein was exchanged into storage buffer (20 mM PBS, pH 7.5) and stored at −80 °C until use.

### 2.4. The Lytic Activity Determination of Endolysin LysSE24

The lytic activity of endolysin LysSE24 was quantitatively assayed using improved Mikoulinskaia turbidity method with slight modification [30]. *Salmonella* Enteritidis ATCC13076 was inoculated in TSB-YE medium, and cultured to a logarithmic growth phase. Then, chloroform was added to a final concentration of 0.5% (*v*/*v*) and kept for 20 min. The cells were centrifuged (8000 r/min, 20 min). The cultures were repeatedly washed with sterile deionized water, and then the cells were collected by centrifugation. The bacterial pellet was resuspended in 50 mM Tris-HCl buffer containing 0.1% Triton X-100 (pH 8.2), and adjusted to an OD_600 nm_ of 0.8~1.0. A total of 50 μL endolysins LysSE24 in different final concentrations (from 50 μM to 0.01 μM) were added to 200 μL of bacterial resuspension. OD_600 nm_ was monitored every 5 min at room temperature with Swiss Tecan M200 PRO multifunctional enzyme labeling instrument. The maximum decreased concentration of OD_600 nm_ within 40 min was selected as the optimum concentration for the subsequent experiments. The same volume of Tris-HCl buffer with 0.1% Triton X-100 instead of endolysins was used as the negative control. All analyses were performed in triplicate using *Salmonella* Enteritidis ATCC13076 as target strain. The mean values were calculated unless otherwise stated.

### 2.5. Biochemical Characterization of Endolysin LysSE24

To investigate the thermal stability of LysSE24, 0.5 μM of each endolysin was incubated at different temperatures (20–90 °C) in a water bath for 30 min followed by a cooling step to room temperature. The lytic activity was then measured by turbidity reduction assay [23].

The influence of pH on the lytic activity of LysSE24 was assessed using chloroform permeabilized cells. These cells were resuspended in a universal pH buffer (20 mmol/L sodium citrate, 20 mmol/L sodium malonate, 20 mmol/L Tris-HCl, and 20 mmol/L glycine), and adjusted to different pH’s between 4.0 and 12.0.

The influences of ionic strength on lysis activity were also determined, respectively. NaCl, KCl, CaCl_2_, and MgCl_2_ were prepared at a final concentration of 0.1–10 mmoL/L The chloroform treated bacteria cells were prepared as mentioned earlier. Then, 50 μL 100 nM of treated endolysins were mixed with 200 μL of the bacterial resuspension. The OD_600 nm_ was monitored every 5 min at room temperature. The lytic activity was calculated using the following equation: 100% × (OD_600 nm_ of control − OD_600 nm_ of the reaction mixture with endolysins)/the initial OD_600 nm_ of the control. All the selected calculated data are the OD_600 nm_ values measured for 40 min.

### 2.6. Antibacterial Activity of LysSE24 in Combination with Outer Membrane Permeabilizers (OMPs)

The antibacterial activity of LysSE24 combined with OMPs was determined as previously reported with slight modifications [31]. *Salmonella* Enteritidis ATCC13076 was inoculated in TSB-YE liquid medium containing 0.6% yeast extract, and cultured to a logarithmic growth phase. Afterward, chloroform was added to a final concentration of 0.5% (*v*/*v*) and kept for 20 min. The bacteria treated with chloroform were centrifuged (8000 r/min, 20 min). The cells were repeatedly washed with sterile deionized water. Prior to assaying the activity, the bacterial pellet was resuspended in 50 mM Tris-HCl buffer (pH 8.2) containing the different OMPs (0.1% Triton X-100, 2 mM citric acid and 0.5 mM EDTA, and adjusted to an OD_600 nm_ of 0.8~1.0). Then, 50 μL endolysin in different final concentrations were added to 200 μL of bacterial resuspension. OD_600 nm_ was monitored every 5 mins at room temperature. The same volume of Tris-HCl buffer containing different OMPs was used instead of the endolysins for control.

### 2.7. The Lytic Spectrum of LysSE24

For the determination of the antibacterial spectrum of LysSE24, 33 strains were used (Table 1), including 23 strains of drug-resistant *Salmonella*. The relative lytic activity was measured according to the method described in Section 2.4 and calculated using the equation in Section 2.5.

### 2.8. Observation by Transmission Electron Microscopic (TEM)

TEM was used to visualize the effect of LysSE24 on bacterial cells. *Salmonella* Enteritis ATCC13076 was inoculated in TSB-YE liquid medium containing 0.6% yeast extract, and cultured to the logarithmic growth phase. The cells were treated with 0.5 mM EDTA for 20 min. The medium containing EDTA was removed by centrifugation. The cells were washed with sterile water 4 times. Then, 50 mM Tris-HCl buffer containing 0.5 mM EDTA (pH 8.2) was used to adjust the OD_600 nm_ of the bacterial solution to 0.8–1.0. The endolysin LysSE24 was added to a final concentration of 100 nM. For all experiments, 50 mM Tris-HCl of 0.5 mM EDTA (pH8.2) was used as a negative control. After 10 min incubation, cells were fixed with glutaraldehyde at a final concentration of 25% and washed with PBS. Cells were then treated with metaperiodate (1% final concentration, 15 min incubation) and osmium tetroxide plus hexacyanoferrate (1% and 1.5%, respectively; 1 h incubation). Cells were then centrifuged, and the pellets were spun down in microcentrifuge tubes containing melted agar. After solidification of the agar, pellets were embedded in epon for ultra-thin sections (50 nm) preparation, as described. Micrographs were taken with a transmission electron microscope HT7700 (HITACHI, Japan) at an acceleration voltage of 80 kV.

### 2.9. Statistical Analysis

Statistical analysis of the data was performed using the software OriginPro2018 (OriginLab, Northampton, MA, USA). The data were analyzed using the Statistical Package for Social Sciences (SPSS, Chicago, IL, USA) and differences between means were assessed for statistical differences using the Student–Newman–Keuls test (*p* < 0.05). The results show a confidence interval of 95% of the mean. The comparison method of the data is the SNK method (also known as Q test, which is suitable for the comprehensive comparison of the mean of multiple samples) to judge whether there is statistical significance at the test level α = 0.05. All experiments were done in triplicate.

## 3. Results and Discussion

### 3.1. Bioinformatics Analysis of LysSE24

*Salmonella* phage LPSE1 was previously isolated and sequenced [29]. Among 32 ORFs, ORF24, referred to as LysSE24, was identified as a putative endolysin. The physicochemical properties of the LysSE24 sequence were analyzed by the ProtParam tool. LysSE24 consists of 162 amino acids with a molecular weight of 17.4 kD and an isoelectric point of 9.44. The instability coefficient is 34.60 and the hydrophilic coefficient is −0.291. Generally speaking, an instability coefficient of less than 40 indicates that the protein is stable. The negative hydrophilic coefficient indicates that the protein has a certain hydrophilicity [32].

**Figure 1:**
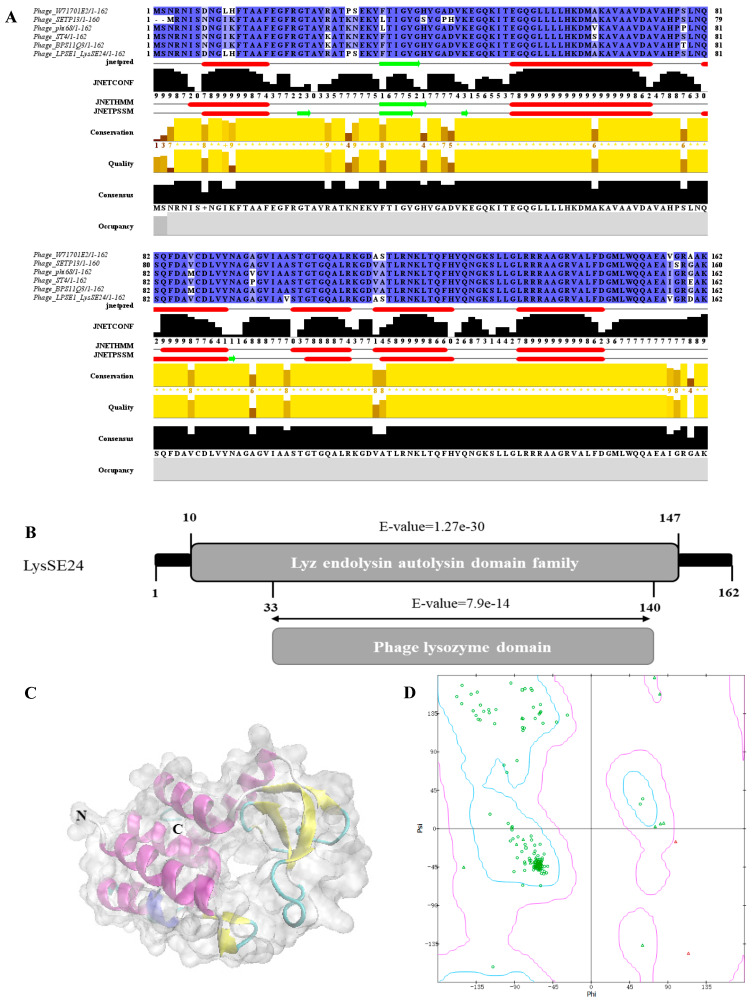
Bioinformatics analysis of LysSE24. (**A**) LysSE24 conserved domain analysis in NCBI Conserved Domain Database (NCMI CDD) and Pfam 32.0. LysSE24 carries out conservative domain search; it belongs to the Lyz endolysin autolysin domain family (Accession cd00737) between10 and 147 aa (E-value 1.27 × 10^−30^), while Pfam predicted that LysSE24 had a phage lysozyme domain (Accession PF00959.19) from 33 to 141 aa (E-value 7.9 × 10^−14^). (**B**) Multiple alignments of amino acid sequences of LPSE1, W71701E2, ST4, BPS11Q3, SETP13, and phi68 endolysins constructed using Clustal-Omega and software Jalview 2.11.0, and LysSE24 secondary structure prediction. The shades of blue in the picture represent the similarity between sequences, while conservation, quality, consensus, and occupancy represent the conservatism, alignment quality, common sequence, and occupation of the sequences, respectively. Jnetpred is the prediction result of Jpred. JNETCONF represents the credibility of secondary structure prediction; the larger the number, the greater the credibility. JNETHMM and JNETPSSM are the results of using HMM and PSSM methods to predict the secondary structure of LysSE24. (**C**) The SWISS-Model server viewed by software VMD 1.9.1 to model the tertiary structure of LysSE24. The N-terminal and C-terminal of the peptide chain are identified, respectively. The helical structure is represented by purple and blue, the sheet structure is represented by yellow, and the irregular curl is represented by green and white, indicating the outer surface shape of the protein LysSE24. (**D**) LysSE24 tertiary structure model used software BIOVIA Discovery Studio 2019 to evaluate the quality of the Ramachandran plot model. The blue area represents the most suitable area, the purple area represents the allowable area, and the red dot represents the unreasonable area.

Sequence alignment analysis of LysSE24 by BLASTP showed that LysSE24 had high similarity with 29 strains of bacteriophage endolysin. The sequences in the result of BLASTP were selected for multi-sequence alignment, which were *Salmonella* phage W71701E2 (similarity 98.77%, E-value 2 × 10^−113^), ST4 (similarity 91.98%, E-value 6 × 10^−100^), BPS11Q3 (similarity 91.98%, E-value 7 × 10^−98^), phi68 (similarity 91.36%, E-value 4 × 10^−97^), and SETP13 (similarity 90.57%, E-value 4 × 10^−92^) (Figure 1A). The LysSE24 sequence was conserved and had high homology with other *Salmonella* bacteriophages. Subsequently, the conserved domain of LysSE24 was analyzed by NCBI CDD and Pfam 32.0. According to NCBI CDD prediction, it belonged to the Lyz endolysin autolysin domain family (Accession cd00737), between 10 and 147 aa [33]. Pfam predicts that LysSE24 had a phage lysozyme domain (Accession PF00959.19) from 33 to 141 aa (Figure 1B) [34]. The analysis showed that LysSE24 could hydrolyze the 1,4-*β*-bond between *N*-acetyl-d-glucosamine and *N*-acetylmuramic acid in the peptidoglycan heteropolymer of the prokaryotic cell wall, confirming that it is a lysozyme (*N*-acetylmuramidases).

JPred in the software Jalview2.11.0 was used to predict the secondary structure of the amino acid sequence of LysSE24. Meanwhile, JPred can also use HMM and PSSM methods to predict the secondary structure of proteins (Figure 1B). The prediction results of LysSE24 by JPred, HMM, and PSSM are consistent. The secondary structure of LysSE24 is mainly helix and less lamellar structure, so it can be inferred that the lyase has a stable structure [35]. The homologous modeling method is used to simulate the three-level structure of LysSE24 in SWISS-Model. In the modeling results, PDB ID 6et6.1.A was selected as the modeling template. Additionally, LysSE24 and 6et6.1.A seq identities were 33.10% and QMEAN-2.14, respectively. The constructed LysSE24 model was highly reliable (Figure 1C) [36]. The quality of the LysSE24 model was evaluated by a Ramachandran plot and Verify-3D. In the Ramachandran plot, the Gly at positions 42 and 48 are in the unreasonable region, the rest of the amino acids are in the most suitable region and allowable region, and 92.59% of the amino acids are in the most suitable region (Figure 1D). In the Verify-3D assessment, at least 80% of the amino acids scored ≥0.2 in the 3D-1D profile, and LysSE24 had 95.95% of the residues with an averaged 3D-1D score ≥0.2, so the model of LysSE24 was passed [37]. In conclusion, the homologous modeling of LysSE24 was credible.

### 3.2. Large Scale Expression and Purification Endolysin LysSE24

**Figure 2:**
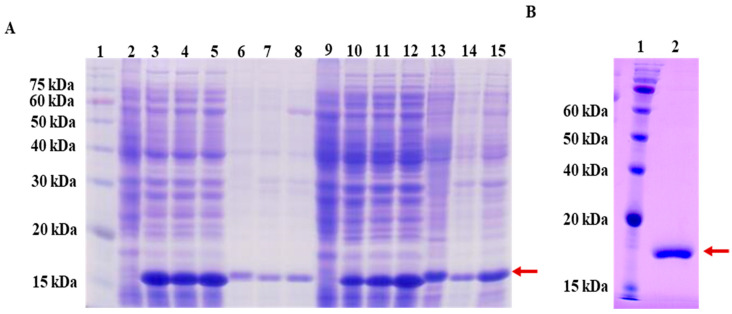
SDS-PAGE analysis of the endolysin LysSE24. (**A**) Lane 1, protein markers; Lane 2, non-induced *E. coli* BL21 culture (negative control); Lane 3, native protein extract (NPE) from *E. coli* BL21 induced for 16 h at 16 °C; Lane 4, NPE from *E. coli* BL21 induced for 4 h at 30 °C; Lane 5, NPE from *E. coli* BL21 induced for 4 h at 37 °C; Lane 6, denatured protein extract (DPE) from *E. coli* BL21 induced for 16 h at 16 °C; Lane 7, DPE from *E. coli* BL21 induced for 4 h at 30 °C; Lane 8, DPE from *E. coli* BL21 induced for 4 h at 37 °C; Lane 9, non-induced *E. coli* C41 culture (negative control); Lane 10, NPE from *E. coli* C41 induced for 16 h at 16 °C; Lane 11, NPE from *E. coli* C41 induced for 4 h at 30 °C; Lane 12, NPE from *E. coli* C41 induced for 4 h at 37 °C; Lane 13, DPE from *E. coli* C41 induced for 16 h at 16 °C; Lane 14, DPE from *E. coli* C41 induced for 4 h at 30 °C; Lane 15, DPE from *E. coli* C41 induced for 4 h at 37 °C; the arrow denotes the target protein; (**B**) Lane 1, protein markers; Lane 2, purified endolysin LysSE24 from *E. coli* BL21 after induction for 16 h at 16 °C; the arrow denotes the target protein.

To optimize the expression of LysSE24, different expression parameters were tested. The expression vectors encoding one possible endolysin were constructed and then transformed into *E. coli* BL21 and *E. coli* C41, respectively. Both NPE and DPE portion collected after induction were subjected to SDS-PAGE. As shown in Figure 2A, new obvious bands at 17 kDa appeared after induction, respectively, which were consistent with the predicted size of LysSE24 through ExPASy (http://web.expasy.org/compute_pi/). For LysSE24, a larger yield was obtained when *E. coli* BL21 was induced at 16 °C for 16 h. After large scale-up expression, LysSE24 in supernatant fractions was purified by a Ni-NTA column, and analyzed by SDS-PAGE. Figure 2B indicated that high-purity LysSE24 was obtained. The purity was about 90% by using the Bradford method, which yielded a soluble protein of 175.5 mg per liter of culture.

### 3.3. The Lytic Activity of the Endolysin LysSE24

**Figure 3:**
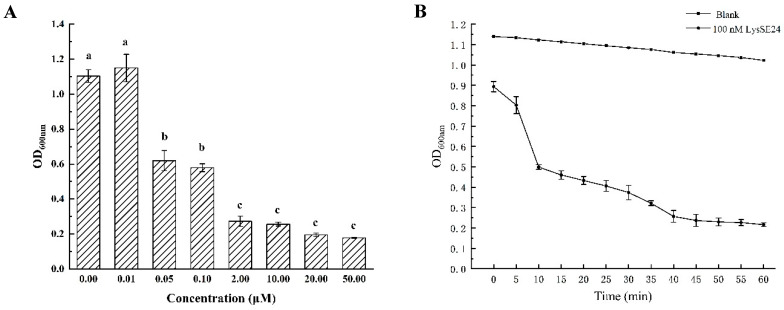
The effect of endolysin LysSE24 on the OD_600nm_ values of host strain *S.* Enteritidis ATCC 13076. (**A**) The effect of different concentrations of LysSE24 on the OD_600nm_ value of *S.* Enteritidis ATCC 13076. The different letters (a, b, c, and d) in the figure indicate significant differences (*p* < 0.05). (**B**) The OD_600nm_ values of *S.* Enteritidis ATCC 13076 was measured every 5 min for a total of 60 min by using 100 nM LysSE24.

The lytic activity of LysSE24 was determined by turbidimetry, as shown in Figure 3. When the final concentration of LysSE24 was from 100 nM to 50 μM for 10 min, the turbidity of *Salmonella* Enteritis ATCC 13076 decreased significantly. Then, 100 nM LysSE24 was selected to act on *Salmonella* and OD_600nm_ was determined every 5 min for a total of 60 min. The results showed that when 100 nM LysSE24 acted on *Salmonella* Enteritis 13076 for 10 min, OD_600nm_ decreased from 1.0 to 0.5 (*p* < 0.05), which could reach half inhibition. Therefore, LysSE24, with the final concentration of 100 nm, was selected as the protein concentration of the follow-up experiment. This result showed that LysSE24 exhibited relatively high lytic activity on *Salmonella* [38,39,40].

### 3.4. Characterization of the Endolysin LysSE24

#### 3.4.1. Temperature Resistance

**Figure 4:**
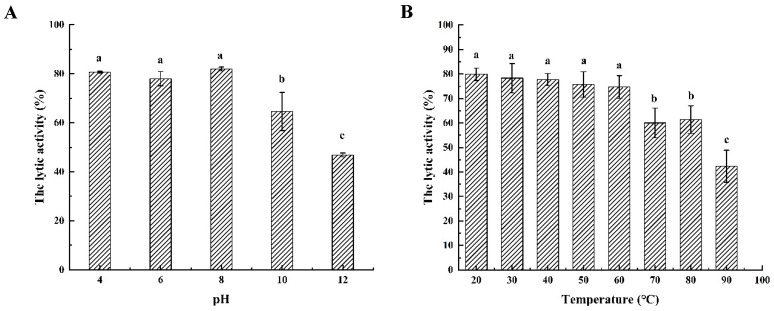
The stability of the endolysin LysSE24. (**A**) Temperature resistance of endolysin LysSE24. (**B**) pH dependence of endolysin LysSE24 enzymatic activity. Calculate the lytic activity by using the formula: 100% × (OD_600 nm_ of control − OD_600 nm_ of the reaction mixture with endolysins)/the initial OD_600 nm_ of the control. The different letters (a, b, c, and d) in the figure indicate significant differences (*p* < 0.05).

To test the characteristics of endolysins, the lytic activity of LysSE24 was investigated at various temperatures. As shown in Figure 4, the lytic activity of LysSE24 was approximately 75% when incubated at 20 to 60 °C for 30 min. The activity is reduced significantly (*p* < 0.05) after 60 °C and reduced slowly at 70 to 90 °C for 30 min. Therefore, LysSE24 is more tolerant of high temperatures than previously reported endolysins, which was similar to endolysins Gp110 and LysPA26. The enzyme was treated at a high temperature for 30 min. The lytic activity was restored after being allowed to stand at room temperature [23,41].

#### 3.4.2. pH Dependence of Endolysin LysSE24 Enzymatic Activity

The dependence of pH on the lytic activity of LySE24 was tested. The endolysin showed high lytic activity at pH 4.0 to 10.0 (Figure 4), which were about 80% and 75%, respectively. When the pH was greater than 10.0, a significant decrease in activity was observed (*p* < 0.05). Therefore, it was speculated that LysSE24 had higher lytic activity under slightly acidic, neutral, and slightly alkaline conditions, and the cleavage rate was higher at pH 8.0. Precipitation occurred at pH 2.0, demonstrating that endolysin could not tolerate a highly acidic environment. Therefore, it was speculated that LysSE24 was an alkalophilic endolysin. The trends of their activities that were affected by pH were similar to endolysins LysIS 075 and LysCER057 [42] but different from the neutrophilic endolysins from Lys68 [43], which showed optimum activity at 7.5 and 7.0, respectively.

#### 3.4.3. The Different Metal Ionic Effect of LysSE24 Activity

**Figure 5:**
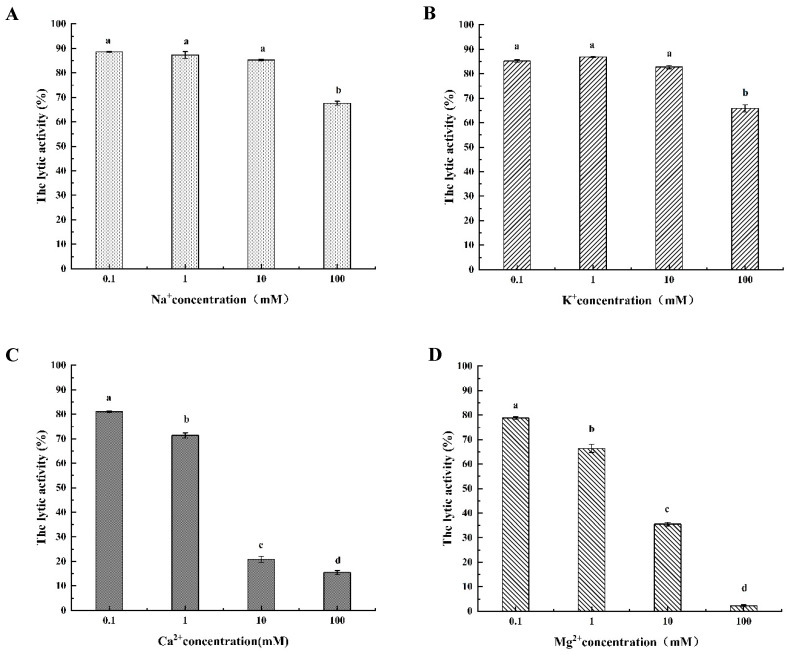
The different metal ionic effects of LysSE24 activity. **A**. Effect of sodium ion on the lytic activity of endolysin LysSE24. **B**. Effect of potassium ion on the Lysis of Endolysin LysSE24. **C**. Effect of calcium ion on lytic activity of endolysin LysSE24. **D**. Effect of magnesium ion on the cleavage of endolysin LysSE24. Calculate the lytic activity by using the formula: 100% × (OD_600 nm_ of control − OD_600 nm_ of the reaction mixture with endolysins)/the initial OD_600 nm_ of the control. The different letters (a, b, c, and d) in the figure indicate significant differences (*p* < 0.05).

The lytic activity of metal ions on LysSE24 was shown in Figure 5. When potassium ions and sodium ions are at 0.1, 1, and 10 mM, there is no significant inhibitory effect on the cleavage activity of LysSE24 (*p* > 0.05). At 100 mM, both potassium and sodium ions showed some inhibition, but not strong inhibition (*p* < 0.05). The inhibitory effect of the calcium and magnesium ions on the lytic activity of LysSE24 was evident at 10 and 100 mM. As the concentration of divalent metal ions increases, the lytic activity of the endolysin LysSE24 decreases, and at 100 mM, the lytic rate is minimized. The results showed that the inhibitory effect of high concentration of univalent metal ion (Na^+^ and K^+^) on the lytic activity of internal endolysin LysSE24 was significantly lower than that of high concentration of divalent metal ion (Ca^2+^ and Mg^2+^; *p* < 0.05). When the concentration of these four metal ions was as high as 10 mmol/L, the lytic activity of endolysin LysSE24 decreased significantly, which is consistent with the endolysins produced by bacteriophages λSA2 and B30 [44]. Divalent metal ions bind to amino acid residue sites on different domains of the endolysin, which resulted in a difference in the activity of the endolysins [45,46]. The binding reaction of the metal ions with LysSE24 needs further study.

### 3.5. Antibacterial Activity of LysSE24 in Combination with OMPs

The thicker outer membrane of Gram-negative bacteria is the main barrier to the lytic function of phage lysins on the peptidoglycan layer. When applied to Gram-negative bacteria alone, endolysins are usually ineffective or inefficient. In the current study, endolysins derived from phage are often combined with an outer membrane permeabilizing agent to enhance antibacterial efficacy. The antibacterial activity of LysSE24 in combination with 0.5 mM EDTA, 2 mM citric acid (CA), or 0.1% (*v*/*v*) Triton X-100 was determined.

**Figure 6:**
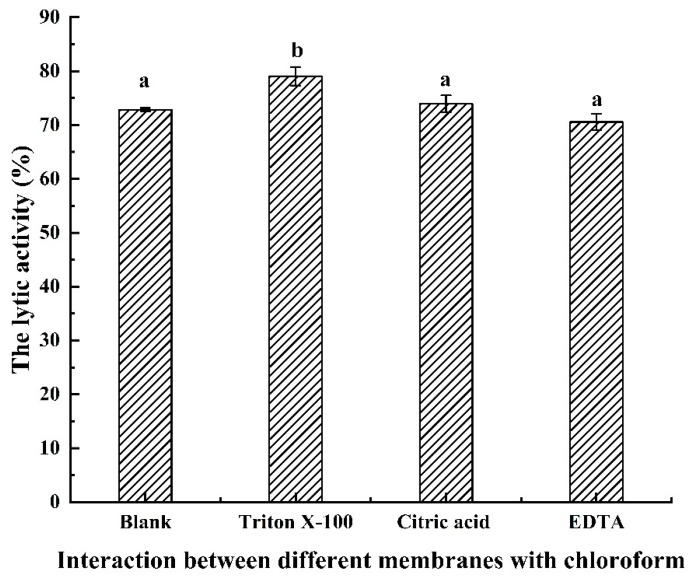
The effect of chloroform combined with three different outer membrance permeabilizers on the lytic ability of LysSE24. Blank: The endolysin LysSE24 (final concentration 100 nM) was added after the cells were treated with chloroform only. The concentrations of the three outer membrance permeabilizers were 0.1% Triton X-100, 2 mM citric acid, and 0.5 mM EDTA. Calculate the lytic activity by using the formula: 100% × (OD_600 nm_ of control − OD_600 nm_ of the reaction mixture with endolysins)/the initial OD_600 nm_ of the control. The different letters (a, b, c, and d) in the figure indicate significant differences (*p* < 0.05).

As shown in Figure 6, the cells were first treated with 0.5% (*v*/*v*) chloroform for 5 min; then, the chloroform was washed away. Additionally, the cells were washed three times with sterile deionized water in the blank group (only endolysin LysSE24 was added). In the 0.5 mM EDTA and 2 mM CA group, the lytic activity rate reached more than 70% after the addition of the endolysin LysSE24. The lytic rate was above 75% in the group added with 0.1% Triton X-100 (*v*/*v*). After combining endolysin LysSE24 with Triton X-100 or 0.5% (*v*/*v*) chloroform, the endolysin LysSE24 showed the strongest antibacterial effect.

Due to the protection of the outer membrane, Gram-negative bacteria have great resistance to exogenous endolysins. Removal of the outer membrane with chloroform could significantly improve the cleavage activity of LysSE24 to host bacteria. Chloroform treatment showed high efficiency in destabilizing the outer membrane and allowed lysine to reach peptidoglycan in turbidity reduction analysis [30,38,39]. In this study, the LysSE24 of 100 nM and the commonly used outer membrane permeating agent reduced the turbidity of chloroform-treated host strains by more than 70% in 10 min, indicating that LysSE24 has rapid and strong lytic activity against *Salmonella* Enteritidis (*p* < 0.05). Outer membrane permeating agents such as chloroform cannot dissolve bacterial cells themselves. However, they can be used as triggers to easily permeate the cell membrane and allow endolysin to spread to the periplasm [47]. An example of the combined application of endolysin and OM permeabilizer was provided by Oliveira et al. They demonstrated that the combination of Lys68 of *Salmonella* bacteriophage with citric acid or malic acid killed *Salmonella* and other Gram-negative organisms [43]. However, some endolysins of bacteriophage show intrinsic antibacterial activity against Gram-negative bacteria without OMPs, such as endolysin of phage CfP1 against *Citrobacter freunii* [48]. However, the exact pattern of these endolysins is unclear and deserves further study [27]. In the future, it is possible to develop a biocide combining OMPs with endolysins, and apply this new biocide to agricultural and food safety control to control the contamination of food-borne Gram-negative bacteria, especially *Salmonella*.

### 3.6. The Lytic Spectrum of the Endolysin LysSE24

**Figure 7:**
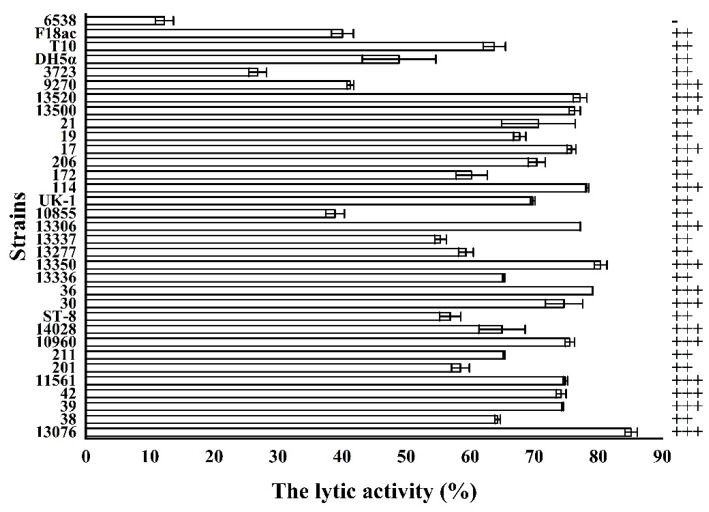
The lytic spectrum of the endolysin LysSE24. Calculate the lytic activity by using the formula 100% × (OD_600 nm_ of control − OD_600 nm_ of the reaction mixture with endolysins)/the initial OD_600nm_ of the control. Lytic activity between 20% to 30%, “+”; lytic activity between 31% to 70%, “++”; lytic activity between 71% to 100%, “+++”; lytic activity lower than 20%, “–”.

The 33 strains were utilized to determine the lytic spectrum of LysSE24 (Table 1). As shown in Figure 7, LysSE24 was able to lytic all the Gram-negative strains listed, i.e., capable of lysing 32 of them (32/33 = 96.97%). Among them, LysSE24 was able to lytic 23 strains of different Gram-negative bacteria, including *Salmonella* Typhimurium, *Salmonella* Enteritidis, *Salmonella* Argora, *Salmonella* Indiana, *Salmonella* Anatum, *Salmonella* Dublin, and *E. coli*. They showed a better lytic effect on different serotypes of drug-resistant *Salmonella*. The endolysin LysSE24 showed no lytic effect on one Gram-positive strain. Similarly, endolysin LysPA26 and LySMP from bacteriophages also showed a broader lytic spectrum than their entire bacteriophages [41,49]. In general, endolysin is genus- or species-specific, that is, endolysin in *Salmonella* phage may only be active against different serotypes of *Salmonella*. However, in a few cases, this endolysin may be active against other genera, even against Gram-positive and Gram-negative strains. LysSE24 can lytic *Salmonella* and *E. coli*. At present, due to the continuous growth of bacterial drug resistance, there is an urgent need to find substitutes for antibiotics to control pathogenic bacteria. In this study, 100 nM LysSE24 can cleave a panel of 23 tested multidrug-resistant *Salmonella* strains, and it will have a prospect as a biological agent to control multidrug-resistant bacteria. More importantly, until now, no specific resistance of bacteria to endolysins has been reported [50].

### 3.7. Observation by Transmission Electron Microscopy (TEM)

**Figure 8:**
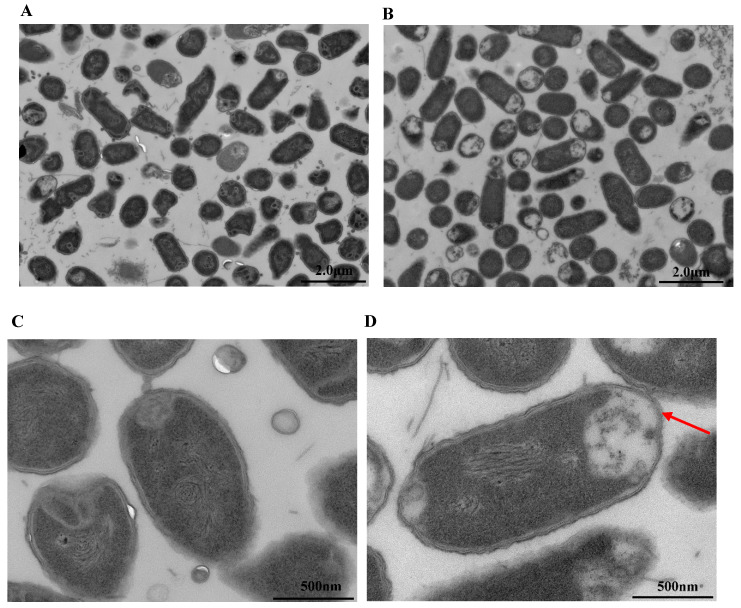
Transmission electron micrograph (TEM) of *S*. Enteritidis ATCC 13076. (**A**,**C**) Blank group: The cells were treated with 0.5 mM EDTA for 20 min and amplified by transmission electron microscopy for 0.2 μm and 500 nm. (**B**,**D**) Experimental group: The cells were treated with 0.5 mM EDTA for 20 min and 100 nM endolysin LysSE24, amplified by transmission electron microscopy for 0.2 μm and 500 nm.

The effect of LysSE24 on *S.* Enteritidis ATCC 13076 structure was visualized by TEM. As shown in Figure 8, exposure to 100 nM of endolysin LysSE24 led to drastic changes in intracellular density and to the disintegration of the cell wall and the cell membrane. As similar as endolysin LytSD and PlyE146, it can be concluded that the strain suffered severe damage after being treated by endolysin LysSE24, which was not only reflected in the cell wall but also on the cell membrane [27,51].

## 4. Conclusions

In this study, we confirmed the lytic activity of an endolysin homologue from LPSE1, a newly isolated bacteriophage infecting *Salmonella* Enteritidis. Endolysin LysSE24 has a lysozyme-like superfamily domain and its characterization was verified. Overall, this globular endolysin exhibits a broad and high activity against Gram-negative pathogens, especially several multidrug-resistant *Salmonella* strains in vitro. Further studies can be developed to improve its bactericidal activity without the need for outer membrane-destabilizing agents by synthetic biology methods.

## Figures and Tables

**Table 1:** Bacterial strains used in the study.

Number	Strain	Strain Type	Source	Drug-Resistance	
1	13076	*Salmonella* Enteritidis	ATCC ^a^	-	
2	38	*Salmonella* Enteritidis	Lab Collection	+	Amoxycillin, Ampicillin, Nalidixic acid, Sulfonamides
3	39	*Salmonella* Enteritidis	Lab Collection	+	Amoxycillin, Ampicillin, Nalidixic acid
4	42	*Salmonella* Enteritidis	Lab Collection	+	Nalidixic acid
5	11561	*Salmonella* Enteritidis	Lab Collection	+	Ampicillin, Ceftriaxone, Streptomycin, Nalidixic acid, Trimethoprim-sulfamethoxazole, Azithromycin, Fosfomycin
6	201	*Salmonella* Enteritidis	Lab Collection	+	Ampicillin, Amoxycillin/clavulanic acid, Gentamicin, Spectinomycin, Tetracycline, Sulphafurazole, Apramycin
7	211	*Salmonella* Enteritidis	Lab Collection	+	Enrofloxacin, Ofloxacin, Apramycin, Colistin
8	10960	*Salmonella* Enteritidis	Lab Collection	+	Tetracycline, Nalidixic acid, Fosfomycin
9	14028	*Salmonella* Typhimurium	ATCC	-	
10	ST-8	*Salmonella* Typhimurium	Lab Collection	-	
11	30	*Salmonella* Typhimurium	Lab Collection	+	Amoxycillin, Ampicillin, Chloramphenicol, Nalidixic acid, Gentamicin, Nitrofurantoin, Sulfonanides, Tetracycline
12	36	*Salmonella* Typhimurium	Lab Collection	+	Amoxycillin, Ampicillin, Chloramphenicol, Straptomycin, Sulfonanides, Tetracycline
13	13336	*Salmonella* Typhimurium	Lab Collection	+	Ampicillin, Straptomycin, Gentamicin, Kanamycin, Chloramphenicol, Nalidixic acid, Ofloxacin, Ciprofloxacin, Sulphafurazole, Trimethoprim- Sulfamethoxazole,
14	13350	*Salmonella* Typhimurium	Lab Collection	+	Ampicillin, Streptomycin, Gentamicin, Kanamycin, Chloramphenicol, Tetracycline, Nalidixic acid, Ofloxacin, Ciprofloxacin, Sulphafurazole, Trimethoprim-sulfamethoxazole,
15	13277	*Salmonella* Typhimurium	Lab Collection	+	Ampicillin, Streptomycin, Gentamicin, Kanamycin, Chloramphenicol, Tetracycline, Nalidixic acid, Ofloxacin, Ciprofloxacin, Sulphafurazole, Trimethoprim-sulfamethoxazole,
16	13337	*Salmonella* Typhimurium	Lab Collection	+	Ampicillin Streptomycin, Gentamicin, Kanamycin, Chloramphenicol, Tetracycline, Nalidixic acid, Ofloxacin, Ciprofloxacin, Sulphafurazole, Trimethoprim-sulfamethoxazole,
17	13306	*Salmonella* Typhimurium	Lab Collection	+	Ampicillin, Streptomycin, Gentamicin, Kanamycin, Chloramphenicol, Tetracycline, Nalidixic acid, Ofloxacin, Ciprofloxacin, Sulphafurazole, Trimethoprim-sulfamethoxazole,
18	10855	*Salmonella* Typhimurium	Lab Collection	+	Ampicillin, Ceftriaxone, Ceftiofur, Kanamycin, Streptomycin, Tetracycline, Doxycycline, Ofloxacin, Sulphafurazole
19	UK-1	*Salmonella* Typhimurium	Lab Collection	-	
20	114	*Salmonella* Typhimurium	Lab Collection	+	Ampicillin, Amoxycillin/Clavulanic acid, Gentamicin, Spectinomycin, Tetracycline, Sulphafurazole, Trimethoprim-sulfamethoxazole, Apramycin, Colistin, Mequindox
21	172	*Salmonella* Typhimurium	Lab Collection	+	Amoxycillin/Clavulanic acid, Florfenicol, Sulphafurazole, Trimethoprim-sulfamethoxazole, Ceftiofur, Ceftazidime, Meropenem, Apramycin
22	206	*Salmonella* Typhimurium	Lab Collection	+	Sulphafurazole, Trimethoprim-sulfamethoxazole, Colistin, Mequindox
23	17	*Salmonella* Argora	Lab Collection	+	Amoxycillin, Ampicillin, Streptomycin, Sulfonamides, Tetracycline
24	19	*Salmonella* Argora	Lab Collection	+	Amoxycillin, Ampicillin, Cephazolin, Nalidixic acid
25	21	*Salmonella* Argora	Lab Collection	+	Chloramphenicol
26	13500	*Salmonella* Indiana	Lab Collection	+	Ampicillin, Amoxycillin/Clavulanic Acid, Streptomycin, Tetracycline, Ceftriaxone, Trimethoprim-sulfamethoxazole, Nalidixic acid, Ciprofloxacin, Fosfomycin, Chloramphenicol, Azithromycin
27	13520	*Salmonella* Indiana	Lab Collection	+	Ampicillin, Amoxycillin/clavulanic acid, Amikacin, Streptomycin, Tetracycline, Ceftriaxone, Trimethoprim-sulfamethoxazole, Nalidixic acid, Ciprofloxacin, Fosfomycin, Chloramphenicol, Azithromycin
28	9270	*Salmonella* Anatum	CMCC ^b^	-	
29	3723	*Salmonella* Dublin	Lab Collection	-	
30	DH5α	*Escherichia coli*	Lab Collection	-	
31	T10	*Escherichia coli*	Lab Collection	-	
32	F18ac	*Escherichia coli*	Lab Collection	-	
33	6538	*Staphylococcus aureus*	Lab Collection	-	

+, Strain was drug-resistant; -, strain was not drug-resistant. ^a^ ATCC, American Type Culture Collection. ^b^ CMCC, Center for Medical Culture Collections.
